# Bat Species Comparisons Based on External Morphology: A Test of Traditional versus Geometric Morphometric Approaches

**DOI:** 10.1371/journal.pone.0127043

**Published:** 2015-05-12

**Authors:** Daniela A. Schmieder, Hugo A. Benítez, Ivailo M. Borissov, Carmelo Fruciano

**Affiliations:** 1 Sensory Ecology Group, Max Planck Institute for Ornithology, Seewiesen, Germany; 2 Conservation Biology, Institute of Ecology and Evolution, University of Bern, Bern, Switzerland; 3 Faculty of Life Sciences, University of Manchester, Manchester, United Kingdom; 4 Instituto de Alta Investigación, Universidad de Tarapacá, Arica, Chile; 5 Department of Zoology, University of Tel Aviv, Tel Aviv, Israel; 6 Lehrstuhl für Zoologie und Evolutionsbiologie, Department of Biology, University of Konstanz, Konstanz, Germany; 7 Department of Biological, Zoological and Environmental Sciences, University of Catania, Catania, Italy; Brown University, UNITED STATES

## Abstract

External morphology is commonly used to identify bats as well as to investigate flight and foraging behavior, typically relying on simple length and area measures or ratios. However, geometric morphometrics is increasingly used in the biological sciences to analyse variation in shape and discriminate among species and populations. Here we compare the ability of traditional versus geometric morphometric methods in discriminating between closely related bat species – in this case European horseshoe bats (Rhinolophidae, Chiroptera) – based on morphology of the wing, body and tail. In addition to comparing morphometric methods, we used geometric morphometrics to detect interspecies differences as shape changes. Geometric morphometrics yielded improved species discrimination relative to traditional methods. The predicted shape for the variation along the between group principal components revealed that the largest differences between species lay in the extent to which the wing reaches in the direction of the head. This strong trend in interspecific shape variation is associated with size, which we interpret as an evolutionary allometry pattern.

## Introduction

Studies relating bat wing morphology to flight characteristics date back to the beginning of the 20^th^ century [1–4]. Lengths and areas of wings or their parts were typically measured to compare wing morphology among species. Wing loading, the first ratio proposed, is still used for size-independent species comparison [3]. In following decades, further ratios, such as aspect ratio, tip length ratio, tip area ratio and the wingtip shape index were defined [5–7]. Many studies of wing morphology were conducted using these ratios to compare interspecies morphology [5,8–13]. The methods for obtaining raw morphometric variables (e.g. wing area or arm wing area) to construct such ratios often varied among studies, making results difficult to compare (see [5,7,10,11,14–16] for examples). Another problem was the way in which bats were measured. That is, early studies collected measurements on museum specimens [5,7], while later ones relied on wing tracings from live bats [10,12,17,18]. Finally, in recent years, photographs of bats with fully extended wings have been analysed with image programs [19,20]. For a long time, differences in total wing area and shape in relation to flight and foraging performance were emphasized and in most studies the tail was assumed to play a minor role or was not measured independently. Typically, the tail area was included in measurements of the wing area together with the body or parts of the body [5,8,10–13]. However, Schmieder et al. [16], used two ratios to exclusively capture tail morphology and found differences between two similar species.

Since the 80s a new set of morphometric techniques has been established: geometric morphometrics [21–23]. This set of techniques has gained enormous popularity and has been used across a large number of taxa and questions (for a recent review see [22]). These methods have become popular because they permit separation of the size and shape components of morphometric variation. The resulting variables are not redundant and these approaches allow visualization of results in terms of shape changes while retaining the geometric properties of objects throughout the analysis [24]. Moreover, geometric morphometrics allows quantifying changes in the position of anatomical structures relative to one another, which sometimes are not captured by linear morphometric techniques. Contrasting this popularity in studies of other taxa, geometric morphometrics has had limited use in studies of external morphology in bats (but see [25,26]). In the present study, we quantitatively compared the ability of traditional and geometric morphometric methods to discriminate among species of bats based on external morphology. If geometric morphometrics proves to be better in capturing differences in bats’ external morphology, this method could be a helpful tool where traditional morphometrics is limited (i.e. when traditional morphometrics cannot be used to discriminate among species or when shape is itself of interest). We quantitatively compared the different methods by using classification rates in discriminant analysis and we focused on bat species that are closely related and known to be very similar in morphology. We, therefore, set out to analyse the differences in morphology of the five European horseshoe bat species (Rhinolophidae, Chiroptera). These species sometimes overlap in size and they are very similar in morphology [5,13,27–29]. The smallest European horseshoe bat is *Rhinolophus hipposideros* Bechstein, 1800 and the largest is *Rhinolophus ferrumequinum* Schreber, 1774. The other three species: *Rhinolophus blasii* Peters, 1866, *Rhinolophus euryal*e Blasius, 185*3* and *Rhinolophus mehely*i Matschie, 1901 are of intermediate size and sometimes difficult to differentiate. European horseshoe bats do not only overlap in size (especially the intermediate species). In fact, all five species are known to forage in or close to vegetation [30–37] and belong to the same foraging guild—the flutter detecting foragers [38]. In south-eastern Europe all five species occur sympatrically [34] and can partly overlap in diet (e.g. moths [34,39–41]) and hunting strategies (foraging on the wing, foraging from perches and foraging on the ground) [34,42,43]. The species choose similar summer roosting places like caves (in the Mediterranean regions) or roof spaces and other parts within buildings [30,34,36,37]. Although the phylogenetic relationships of this group are not fully resolved, all phylogenies published to date agree in considering *R*. *mehelyi* and *R*. *euryale* to be closely-related [44–46]. *R*. *hipposideros* seems most distantly related to the other four horseshoe bat species and *R*. *blasii* is considered as a sister group of *R*. *ferrumequinum* [47].

## Materials and Methods

### Ethics Statement

Capture and handling of bats was in accordance with recommendations of the Canadian Council on Animal Care on bats [48] and the EUROBATS Resolution [49] and was licensed by the responsible Bulgarian authorities (MOEWSofia and RIOSV-Ruse, field permit numbers 297/09.03.2011, 465/29.06.2012, 554/20.01.2014). The mentioned field permits authorized us to capture and measure (including taping of bats for making wing pictures) the studied species at the differing capture sites (S1 Table). Officials from the Bulgarian Ministry of Environment and Water (MOEW) inspected our work in accordance with Section 8, Article 23, Paragraph 3 and 4 of the Bulgarian Biodiversity Law. According to Bulgarian laws no further ethical approval by a committee is required for a non-invasive study. These procedures were not part of a routine care or monitoring project. No bats were harmed. After the experiments all bats were released in good health at their respective capture sites.

### Animals

We caught bats in a harp trap (Faunatech, Victoria, Australia), mist nets (Ecotone, Sopot, Poland) or hand nets at the entrances of caves, in or near abandoned buildings in north-eastern, central and southern Bulgaria (maximal distance between capture sites approx. 260 km, S1 Table) between May and September in 2011, 2012 and 2014. We identified the five European horseshoe bat species using an identification key [50]. Only adult male bats with no wing injuries were used for wing pictures. We analysed pictures of 6 *Rhinolophus hipposideros*, 7 *Rhinolophus blasii*, 22 *Rhinolophus euryale*, 20 *Rhinolophus mehelyi* and 21 *Rhinolophus ferrumequinum*.

### Wing pictures

Wing photographs were taken by fixing each individual with its ventral side held firmly against the board of a copy stand (custom-made, Max-Planck Institute for Ornithology, Seewiesen, Germany). The board of the copy stand was covered with graph paper and transparent self-adhesive cover film pasted on top of it. The wings and tail membrane were carefully extended (starting with the right wing, then the left wing and ending with the tail membrane) with the largest possible stretching of the wing and tail membrane and fixed each time with transparent adhesive tape (width 19 mm) to the board. For the standardization of wing position, we have considered that the upper arm was at a 90° angle to the midline of the body. Before taking pictures, we ensured that the wings and the tail were properly fixed and that no movement of the fixated body parts was possible. In the rare cases where movement of fixated body parts occurred, we readjusted and re-fixated the wing or tail before taking pictures. While fixating the bat, the head was covered with a black cotton cloth to calm the animal down and to reduce attempts of the bat to move. Each individual was fixed two times to reduce measurement error due to fixation. For each fixation, we took several digital photos with a digital camera (12 megapixel, DMC-TZ10, Panasonic, Ōsaka, Japan) mounted on the copy stand at a height of 47 cm. The tape was then carefully removed without injury. From each of the 76 individuals, we chose the three best pictures (one or two per fixation) which were then used in downstream traditional and geometric morphometric analyses. A picture was chosen as best picture if there was no blurriness in the picture, the wings were fixated symmetrically, the wing and tail membranes were fully extended and the head was straight and pointed towards the plate.

### Measurements

**Fig 1:**
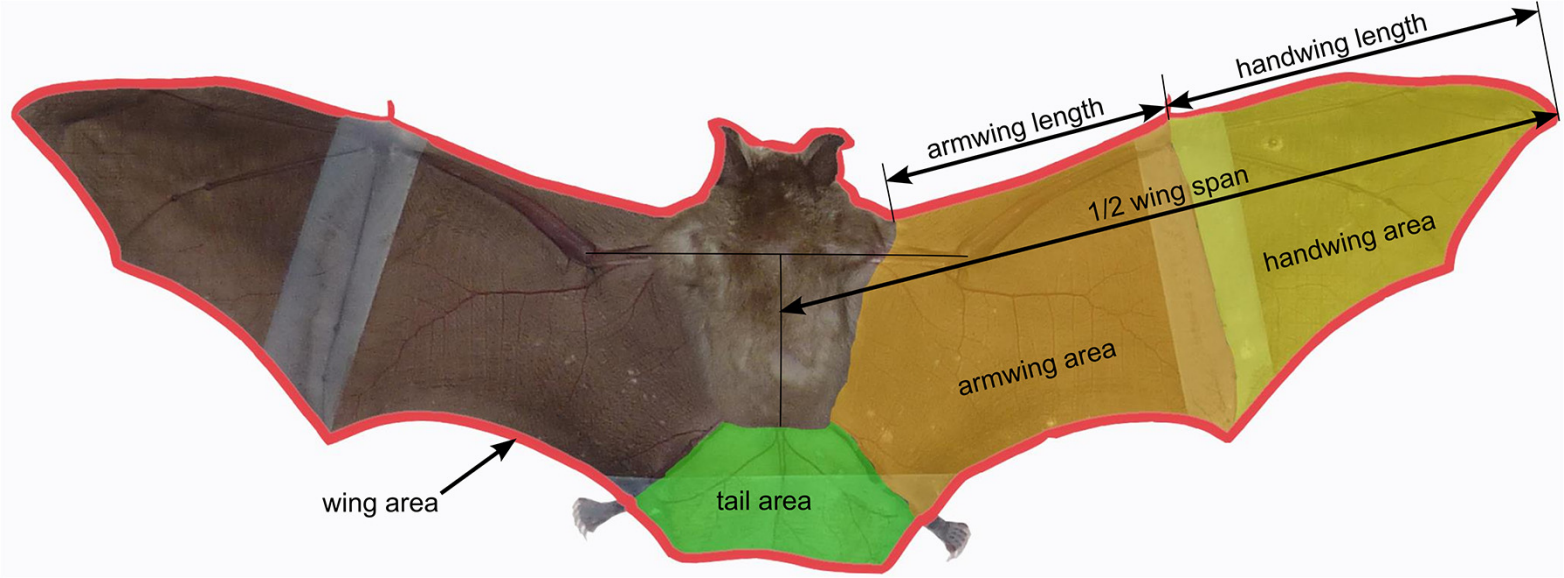
Length and area measurements taken for each analysed wing photograph. These measures were used for methods 1 and 2.

**Fig 2:**
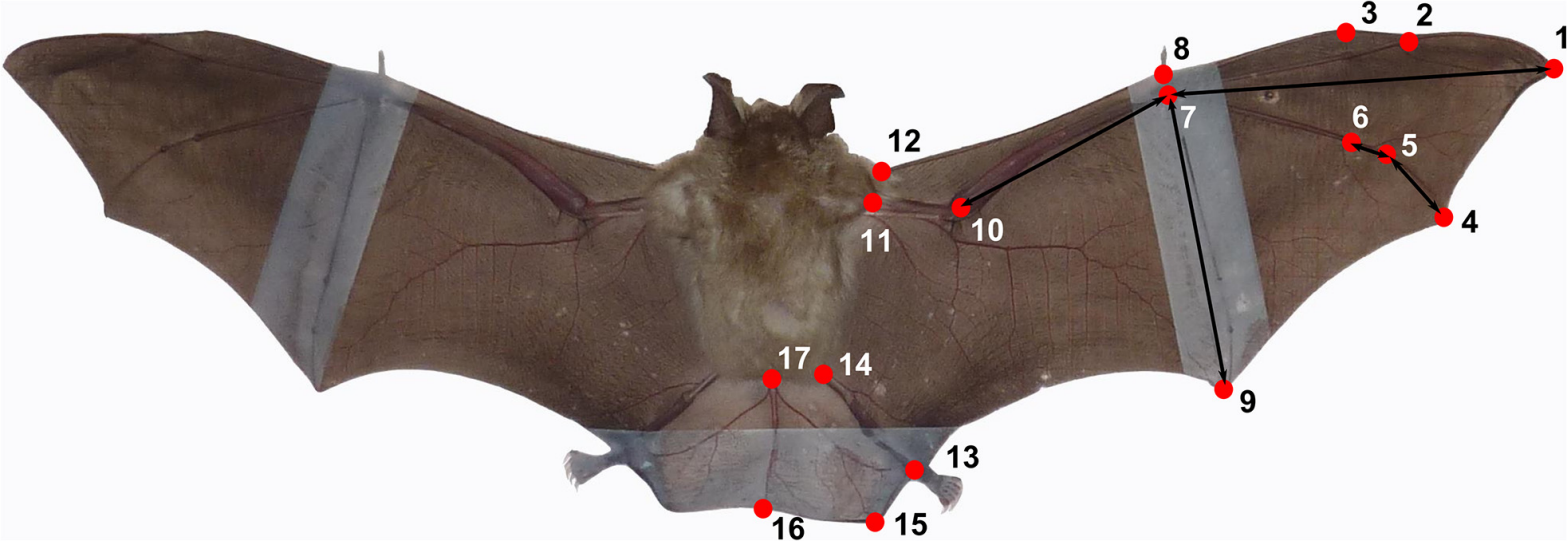
Landmarks used to generate data for methods 3 and 4. All landmarks were used in the geometric morphometric approach (method 4). Arrows show the linear distances that were taken for method 3.

For traditional morphometrics, we measured the right wing, tail and body to obtain multiple lengths (hand wing length, arm wing length, wing span) and areas (arm wing area, hand wing area, tail area and wing area) (Fig 1), using an image processing program (Adobe Photoshop, version 13.0.1, Adobe Systems, San Jose, USA). We also digitized 17 landmarks on the right side of each specimen (Fig 2), using tpsDig [51]. From landmark coordinates, we obtained using the program TMorphGen6 of the IMP package [52], linear distances between the landmarks 1 and 7 (3^rd^ digit), 7 and 9 (5^th^ digit), 5 and 6 (first phalanx of 4^th^ digit) as well as 4 and 5 (second phalanx of 4^th^ digit). For reduction of measurement error in geometric morphometrics, for each bat we subjectively determined and digitized the best two pictures of the best fixation and the best picture of the other fixation. We quantified the measurement error present in the dataset obtained with the above-mentioned experimental design using a Procrustes ANOVA [53], which showed that measurement error was small relative to the variation among individuals and among species (S2 Table). The resulting coordinates were then averaged—thus further reducing measurement error [54–56]—for each bat after a generalized Procrustes analysis [57] in MorphoJ [58]. As a preliminary MANCOVA showed that the interaction between species and centroid size was not significant, residuals of a pooled within-group regression of shape on centroid size (accounting for 16.08% of total variance) were obtained to take into account intra-species allometry and these were used in subsequent analyses.

### Comparison of morphometric methods in species discrimination

In this study we compared four morphometric methods for their ability in discriminating bat species based on external morphology.

The first method involved ratios and other measures that are thought to be “size-independent” (i.e. corrected for allometry): tip length ratio, tip area ratio, wing tip shape index, aspect ratio and wing loading [5] (S3 Table). The second method includes measures from the first method except that wing loading is replaced by relative wing loading (which is less dependent on size) and that the tail-to-wing area ratio is added to the other variables [5,9,16]. In the third method we followed Dietz and colleagues [27], using residuals of regression on forearm length for each of the length measurements of digits 3 and 5 and also the first and second phalanges of the fourth digit. These three methods represent the traditional morphometric methods most commonly used to analyse external bat morphology of European horseshoe bats. In a fourth and final method we employed geometric morphometrics using the set of landmarks defined above. We used landmarks only on the right wings to maintain consistency with the other methods and because preliminary analyses on a subset of the specimens showed a lower measurement error due to fixation, as compared to a symmetric configuration of landmarks on both sides of the bat.

To compare methods, we used the correct classification rate of discriminant analysis estimated using a leave-one-out cross-validation procedure. We obtained discriminant analyses and correct classification rates both for pairwise comparisons among species and using a single discriminant analysis on all the species at the same time (canonical variate analysis). We obtained discriminant functions and correct classification rates for the pairwise comparisons among species using geometric morphometric data (method 4) in MorphoJ. All the other discriminant functions and correct classification rates were computed in SPSS (Version 21.0, IBM Corp. Armonk, NY). Given that linear discriminant analysis is known to have artefactually high classification rates at increasing number of dimensions [59], for the geometric morphometric dataset, we also performed discriminant analyses on, respectively, the first two, three, ten, seventeen and twenty-five principal components. The first twenty-five principal components were chosen performing in the R package nFactors [60] the Anderson's test [61], as suggested by Mitteroecker and Bookstein [59] for dimensionality reduction prior to discriminant analyses. The first two, three, ten and seventeen principal components were chosen arbitrarily as lower numbers of principal components.

To test for the presence of a species signal in the raw geometric morphometric data prior to allometric correction, we also performed discriminant analyses on the geometric morphometric dataset obtained from the measurement reduction procedure without subjecting it to the regression-based removal of the allometric component.

### Geometric morphometrics—testing and visualizing differences among species

In addition to the comparison of different morphometric methods, we exploited the advantages of geometric morphometrics by further analysing the geometric morphometric dataset and visualizing differences among species as shape changes. All the analyses were performed on the right-side configurations described above. However, to visualize results we reflected the configurations of points obtained as results [62], thus producing more easily interpretable “bat-like” symmetric displays.

To visualize patterns of variation among species, we used between-group principal component analysis [63]. This method has been suggested to produce ordinations that are preferable to the commonly used scatterplots of canonical variate scores [59] and is increasingly used in geometric morphometric studies [64,65] as the ordinations do not exaggerate the extent of separation between groups. To better interpret variation along the first between-group principal component (bwgPC1)—which was computed based on data after a pooled within-group regression on centroid size and is therefore already corrected for intra-specific allometry—in terms of evolutionary allometry, we regressed bwgPC1 scores on centroid size. We tested for pairwise differences in mean shape among species using the permutational procedure based on Procrustes distances implemented in MorphoJ (10,000 permutations). Differences between species were visualized through wireframe graphs of each species’ mean shape relative to the grand mean.

## Results

### Comparison of morphometric methods in species discrimination

There are clear differences in correct classification rates across the four methods (Table 1 and S4 Table). The poorest classification rate was found for method 1 [5] which uses ratios related to the wing, followed by method 2 [5,9,16] which uses ratios related to wing and tail and method 3 [27] which employs linear measurements on the wing. The latter two methods were rather similar in correct classification rates. Method 4 (data obtained through geometric morphometrics) achieved the greatest success as correct classification was achieved with 94.7% accuracy when comparing all the species (canonical variate analysis) and ranged between 84.6% and 100% in the pairwise comparison (Table 1). Consistent across-methods among-species differences in correct classification were found. For instance, *R*. *hipposideros* showed consistently high correct classification rates in all methods. Correct classification was higher for geometric morphometrics relative to traditional methods also when the geometric morphometric dataset was subjected to dimensionality reduction (i.e. when discriminant analysis was performed on a subset of principal components; S5 Table).

**Table 1:** Cross-validated correct classification rates using traditional and geometric morphometrics.

		Discriminant analysis using all the species (canonical variate analysis)						Discriminant analyses for each pair of species	
Data acquisition	Method	Overall rate	*R*. *hipposideros*	*R*. *blasii*	*R*. *euryale*	*R*. *mehelyi*	*R*. *ferrumequinum*	Average rate	Range of rates
Traditional morphometrics	1	37.0	66.7	28.6	31.8	42.1	31.6	68.6	46.2–89.3
	2	63.0	100	57.1	59.1	64.7	57.1	88.5	58.6–100
	3	67.1	83.3	85.7	72.7	60.0	57.1	88.7	69.0–100
Geometric morphometrics	4	94.7	100	100	86.4	95.0	100	95.3	84.6–100

Correct classification rates for each pairwise comparison are provided in S3 Table.

Discriminant analyses on the geometric morphometric dataset containing allometric variation produced lower correct classification rates when compared to the geometric morphometric dataset obtained after allometric correction. However, correct classification rates in the former case were still higher than the ones obtained using a traditional morphometric dataset.

In fact, the cross-validated correct classification rate for the CVA on the geometric morphometric dataset containing allometric variation was 93.4% (the same percentage of correct classification is obtained both using the full-dimensional space and using the first 25 principal components) and on average 91.63% in pairwise comparisons (range 76.92–100%). This shows that bat species could be discriminated with geometric morphometrics even in the presence of significant allometry.

### Geometric morphometrics—testing and visualizing differences among species

**Fig 3:**
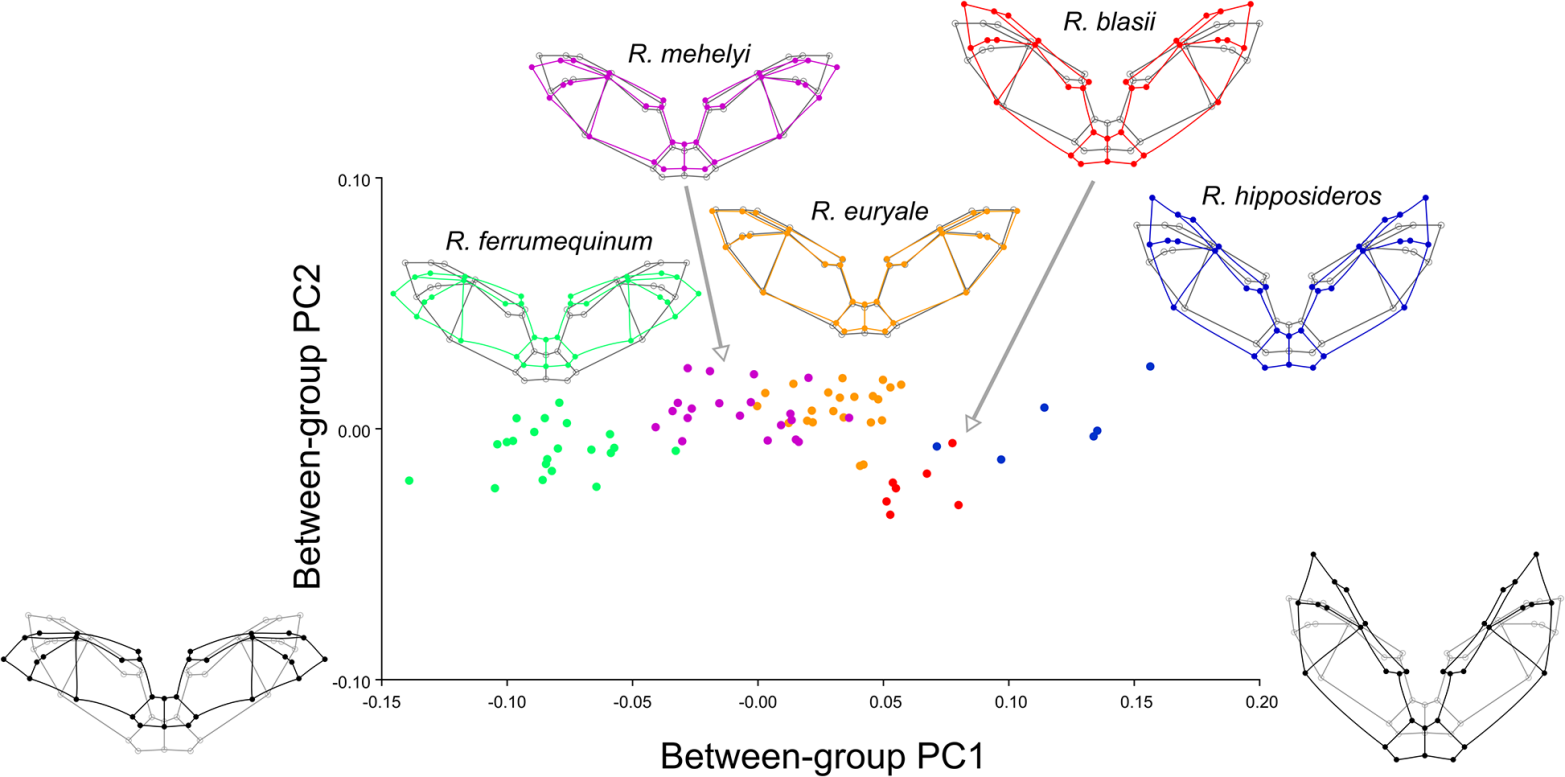
Between-group principal component analysis and average species shapes. Scatterplot of the scores along the first two between-group principal components. Overlaid, predicted shape changes along the first between-group principal component and average shape of each species. Points in the scatterplot are color-coded as the average shapes. In the plots of average species shape, the grand average shape is depicted in grey.

The first between-group principal component accounts for most (91.03%) of the shape variation in the dataset. The second principal component accounted for a mere 4.26% percent of total variance. Interestingly, the different species show little overlap in the scatterplot of the first two between-group principal components (bwgPC), and variation along bwgPC1 mirrors inter-specific variation in body size as species with lower scores on bwgPC1 are larger (Fig 3). This is confirmed by the regression of bwgPC1 scores (which are already corrected for intra-specific allometry) on centroid size, which is significant (p<0.0001) and accounts for 83% of the variation in bwgPC1 scores. Predicted shape for the variation along bwgPC1 (Fig 3) revealed that the largest differences between species lay in the extent to which the wing reaches in the direction of the head. Not much variation among species was present along bwgPC2, with the only exception that *R*. *blasii* has, on average, lower scores along this axis. Considering the low amount of variance explained by bwgPC2 and the fact that it is constructed, by definition, to be orthogonal to bwgPC1, differences along this direction are difficult to interpret and, possibly, of little biological significance. *R*. *mehelyi* and *R*. *euryale* showed the largest level of overlap in the scatterplot.

Permutation tests of difference in average shape were significant across all pairwise comparisons (Table 2). The lowest Procrustes distance was found between *R*. *mehelyi* and *R*. *euryale*, as suggested by their close position in the scatterplot of the scores on the first two between-group principal components.

**Table 2:** Pairwise Procrustes distances among horseshoe bat species (above the diagonal) and *p*-values for the null hypothesis of equal means (below the diagonal).

	*R*. *hipposideros*	*R*. *blasii*	*R*. *euryale*	*R*. *mehelyi*	*R*. *ferrumequinum*
***R*. *hipposideros***	-	0.0656	0.0889	0.1278	0.2005
***R*. *blasii***	0.0003	-	0.0439	0.0760	0.1472
***R*. *euryale***	<.0001	<.0001	-	0.0418	0.1180
***R*. *mehelyi***	<.0001	<.0001	<.0001	-	0.0807
***R*. *ferrumequinum***	<.0001	<.0001	<.0001	<.0001	-

A narrative description of the differences of each species average shape relative to the overall average shape is provided in Table 3 and documents extensive variation in arm-wing, hand-wing, body and tail regions.

**Table 3:** Overview of species differences found with geometric morphometrics.

Species	Overall comparison to average shape	Handwing region	Armwing region	Body	Tail
***R*. *hipposideros***	wing reaches farther in cranial direction	LM 1 and 4 are farther apart resulting in LM 1 being shifted more in cranial direction handwing slightly longer	armwing between LM 13 and 9 enlarged, between LM 8 and 9 broader	shoulder region broader	enlarged tail area and tail longer
***R*. *blasii***	wing reaches farther in cranial direction	handwing slightly longer	armwing between LM 13 and 9 enlarged, Propatagium slightly enlarged, between LM 8 and 9 broader	body longer	shorter tail, enlarged (lateral direction) tail area
***R*. *euryale***	very similar to average shape	normal	normal	normal	smaller tail area
***R*. *mehelyi***	wing reaches less far in cranial direction	normal	armwing between LM13 and 9 shorter	normal	smaller tail area
***R*. *ferrumequinum***	wing reaches less far in cranial direction	handwing between LM 9 and 4 and between 4 and 1 shorter	armwing between LM 13 and 9 shorter, between LM 12 and 8 slightly longer	slightly shorter, in shoulder region broader	slightly longer tail

## Discussion

We compared the ability of four multivariate approaches to discriminate between morphologically similar, closely related species of European horseshoe bats. Landmark-based geometric morphometrics performed best at species discrimination—as measured by its highest levels of correct classification in discriminant analysis. This is not surprising since geometric morphometrics has long been suggested as particularly useful in detecting even relatively small, localized changes in shape [26], making this approach particularly useful in intraspecific studies [66]. Our results suggest that geometric morphometric analyses are as useful in bats as in other groups.

This approach is not practical for rapid species identification in the field. However, it can be used to find anatomical regions where linear measures for species identification in the field should be taken, e.g. for other bat species where no field identification keys exist. Perhaps most importantly, when external morphology and species discrimination are themselves of interest geometric morphometric methods may be especially helpful.

Using geometric morphometrics, we found interspecific differences in horseshoe bats that were not detected by previously used methods. What is more interesting is that we were able to identify strong trends in interspecific shape variation associated with size. In fact, by taking into account allometric variation using a pooled within-group regression, we removed intraspecific allometric variation but not interspecific size-associated shape changes. We, therefore, conclude that the clear trend observed along the first between-group principal component—which accounts for a very high proportion of total variance—can be interpreted as a pattern of evolutionary allometry. Allometry in bats already has been described for various traits (e.g. [67–71]). The comparison of the elongated fingers of bats compared to other mammals may be the most famous example of allometry in regard to morphology [72]. However, to our knowledge, this is the first study to describe evolutionary allometry of external wing morphology in a detailed way and across a group of closely-related bat species.

When considering the functional implication of the shape variation we documented in horseshoe bats, we speculate that a wing reaching farther toward the head—i.e. moving in the positive direction of bwgPC1 (Fig 3)—might be advantageous for flight in dense vegetation. Incidents when bats touch obstacles while wings are positioned in front (ahead of the body centre) might be easier to cope with and therefore less risky. Furthermore it may be easier for bats to evaluate their ability to fly through a specifically narrow spot as well as increasing their manoeuvrability. Especially *R*. *hipposideros* and *R*. *euryale*—which have wings reaching farther towards the head—forage regularly in dense vegetation [30,33–35,73,74]. The foraging behaviour of *R*. *blasii* is less studied, but it is known to forage close to shrubs and hedges [34]. In contrast, the larger species *R*. *mehelyi* and *R*. *ferrumequinum* are at the negative extreme of our bwgPC1 (Fig 3 and Table 3) and these species spend more time in less-cluttered habitat foraging above or along vegetation (e.g. pastures, hedges or arable land) [28,34,37]. Furthermore, both species frequently hunt from perches (flycatcher style) [29,34]. We assume that, for this foraging behaviour, wings reaching less far toward the head should be energetically more efficient during flight. These assumptions should be tested in a biomechanics experiment since bats flight performance cannot be predicted from wing shape alone [71,75]. We cannot determine to which extent the foraging performance of the studied bat species in different environments might be influenced by wing shape alone as opposed to body size as we have shown that these co-vary across species. Former studies, however, have reported that smaller species have better flight performance close to or within cluttered environments [8,14,16,76].

An interesting possibility to test in the future is that our results may describe a more general phenomenon, i.e. bats foraging in dense vegetation have wings reaching farther towards the head compared to bats foraging in edge or open space. Norberg [69] reported that the wings of some bat species show strong convergence with some bird wings, e.g. Mollosid bats have wings similar to the ones of swifts and swallows. Geometric morphometrics is scarcely used to study wing morphology also in birds (but see [77]). It is, therefore, possible that future geometric morphometric studies on birds will allow further (and more precise) tests of the parallelism across taxa of the relationship between wing shape and its functional significance.

## Supporting Information

S1 Table Locality and method of capture for all individuals used in this study. (PDF)

S2 Table Procrustes ANOVA on repeated mesures of shape in the geometric morphometric dataset. SS = sum of squares; MS = mean squares; df = degrees of freedom.
(PDF)

S3 Table List of all traditional morphometric variables used for the discriminant analysis and description of how the measures were taken and what general functional importance they have for flight performance. (PDF)

S4 Table Cross-validated correct classification rates for each pairwise species comparison across different morphometric methods. Rates are expressed as percentages. Species abbreviations as follows: Rhip = *R*. *hipposideros*, Rbla = *R*. *blasii*, Reur = *R*. *euryale*, Rmeh = *R*. *mehelyi*, Rfer = *R*. *ferrumequinum*.
(PDF)

S5 Table Correct cross-validated classification rates (CV rate) for the geometric morphometric dataset after dimensionality reduction when performing discriminant analysis on multiple groups (canonical variate analysis = CVA) and in pairwise comparison. Species abbreviations as follows: Rhip = *R*. *hipposideros*, Rbla = *R*. *blasii*, Reur = *R*. *euryale*, Rmeh = R. mehelyi, Rfer = *R*. *ferrumequinum*.
(PDF)
