# Supplementary material for: Bat Species Comparisons Based on External Morphology: A Test of Traditional versus Geometric Morphometric Approaches
Source: PLoS One. 2015 May 12;10(5):e0127043. doi: 10.1371/journal.pone.0127043 (PMC4428882; doi:10.1371/journal.pone.0127043)
Supplement: S1 Table — (PDF) [file pone.0127043.s001.pdf]

S1 Table. Locality and method of capture for all individuals used in this study.

Species	ID	Year	District	Latitude	Longitude	Capture site	Method	Field permit number
R. hipposideros	Rhip003	2011	Ruse	43.8	25.9	Rocky niche	handnet	297/09.03.2011
R. hipposideros	Rhip004	2011	Ruse	43.8	25.9	Rocky niche	handnet	297/09.03.2011
R. hipposideros	Rhip001	2014	Gabrovo	42.8	25.5	Stanchov	mist net	554/20.01.2014
R. hipposideros	Rhip003	2014	Lovech	42.9	24.5	Barima	handnet	554/20.01.2014
R. hipposideros	Rhip004	2014	Lovech	42.9	24.5	Barima	handnet	554/20.01.2014
R. hipposideros	Rhip005	2014	Ruse	43.5	25.9	Ostritsa	handnet	554/20.01.2014
R. blasii	Rbla001	2011	Kardzhali	41.4	25.5	Samara	mist net	297/09.03.2011
R. blasii	Rbla002	2011	Kardzhali	41.4	25.5	Samara	mist net	297/09.03.2011
R. blasii	Rbla003	2011	Kardzhali	41.4	25.5	Samara	mist net	297/09.03.2011
R. blasii	Rbla004	2011	Kardzhali	41.4	25.5	Samara	mist net	297/09.03.2011
R. blasii	Rbla005	2011	Kardzhali	41.4	25.5	Samara	mist net	297/09.03.2011
R. blasii	Rbla006	2011	Kardzhali	41.4	25.5	Samara	mist net	297/09.03.2011
R. blasii	Rbla007	2011	Kardzhali	41.4	25.5	Samara	mist net	297/09.03.2011
R. euryale	Reur004	2011	Ruse	43.5	25.9	Orlova Chuka	harp trap	297/09.03.2011
R. euryale	Reur005	2011	Ruse	43.5	25.9	Orlova Chuka	harp trap	297/09.03.2011
R. euryale	Reur006	2011	Ruse	43.5	25.9	Orlova Chuka	harp trap	297/09.03.2011
R. euryale	Reur007	2011	Ruse	43.5	25.9	Orlova Chuka	harp trap	297/09.03.2011
R. euryale	Reur012	2011	Lovech	43.2	25.0	Mandrata	mist net	297/09.03.2011
R. euryale	Reur016	2011	Ruse	43.6	26.0	Zorovitza	mist net	297/09.03.2011
R. euryale	Reur017	2011	Ruse	43.6	26.0	Zorovitza	mist net	297/09.03.2011
R. euryale	Reur001	2012	Ruse	43.5	25.9	Orlova Chuka	harp trap	465/29.06.2012
R. euryale	Reur003	2012	Ruse	43.5	25.9	Orlova Chuka	harp trap	465/29.06.2012
R. euryale	Reur004	2012	Ruse	43.5	25.9	Orlova Chuka	harp trap	465/29.06.2012
R. euryale	Reur006	2012	Ruse	43.5	25.9	Orlova Chuka	harp trap	465/29.06.2012
R. euryale	Reur007	2012	Ruse	43.5	25.9	Orlova Chuka	harp trap	465/29.06.2012
R. euryale	Reur009	2012	Ruse	43.5	25.9	Orlova Chuka	harp trap	465/29.06.2012
R. euryale	Reur001	2014	Ruse	43.5	25.9	Orlova Chuka	harp trap	554/20.01.2014
R. euryale	Reur004	2014	Ruse	43.5	25.9	Orlova Chuka	harp trap	554/20.01.2014
R. euryale	Reur005	2014	Ruse	43.5	25.9	Orlova Chuka	harp trap	554/20.01.2014
R. euryale	Reur006	2014	Ruse	43.5	25.9	Orlova Chuka	harp trap	554/20.01.2014
R. euryale	Reur007	2014	Ruse	43.5	25.9	Orlova Chuka	harp trap	554/20.01.2014
R. euryale	Reur008	2014	Ruse	43.5	25.9	Orlova Chuka	harp trap	554/20.01.2014
R. euryale	Reur009	2014	Ruse	43.5	25.9	Orlova Chuka	harp trap	554/20.01.2014
R. euryale	Reur010	2014	Ruse	43.5	25.9	Orlova Chuka	harp trap	554/20.01.2014
R. euryale	Reur011	2014	Ruse	43.5	25.9	Orlova Chuka	harp trap	554/20.01.2014
R. mehelyi	Rmeh001	2011	Ruse	43.5	25.9	Orlova Chuka	harp trap	297/09.03.2011
R. mehelyi	Rmeh002	2011	Ruse	43.5	25.9	Orlova Chuka	harp trap	297/09.03.2011
R. mehelyi	Rmeh003	2011	Ruse	43.5	25.9	Orlova Chuka	harp trap	297/09.03.2011
R. mehelyi	Rmeh004	2011	Ruse	43.5	25.9	Orlova Chuka	harp trap	297/09.03.2011
R. mehelyi	Rmeh005	2011	Ruse	43.5	25.9	Orlova Chuka	harp trap	297/09.03.2011
R. mehelyi	Rmeh006	2011	Ruse	43.5	25.9	Orlova Chuka	harp trap	297/09.03.2011
R. mehelyi	Rmeh007	2011	Ruse	43.5	25.9	Orlova Chuka	harp trap	297/09.03.2011
R. mehelyi	Rmeh008	2011	Ruse	43.5	25.9	Orlova Chuka	harp trap	297/09.03.2011
R. mehelyi	Rmeh005	2012	Ruse	43.5	25.9	Orlova Chuka	harp trap	465/29.06.2012
R. mehelyi	Rmeh006	2012	Ruse	43.5	25.9	Orlova Chuka	harp trap	465/29.06.2012
R. mehelyi	Rmeh008	2012	Ruse	43.5	25.9	Orlova Chuka	harp trap	465/29.06.2012
R. mehelyi	Rmeh009	2012	Ruse	43.5	25.9	Orlova Chuka	harp trap	465/29.06.2012
R. mehelyi	Rmeh010	2012	Ruse	43.5	25.9	Orlova Chuka	harp trap	465/29.06.2012
R. mehelyi	Rmeh011	2012	Ruse	43.5	25.9	Orlova Chuka	harp trap	465/29.06.2012
R. mehelyi	Rmeh003	2014	Ruse	43.5	25.9	Orlova Chuka	harp trap	554/20.01.2014
R. mehelyi	Rmeh004	2014	Ruse	43.5	25.9	Orlova Chuka	harp trap	554/20.01.2014
R. mehelyi	Rmeh005	2014	Ruse	43.5	25.9	Orlova Chuka	harp trap	554/20.01.2014
R. mehelyi	Rmeh006	2014	Ruse	43.5	25.9	Orlova Chuka	harp trap	554/20.01.2014
R. mehelyi	Rmeh007	2014	Ruse	43.5	25.9	Orlova Chuka	harp trap	554/20.01.2014
R. mehelyi	Rmeh008	2014	Ruse	43.5	25.9	Orlova Chuka	harp trap	554/20.01.2014
R. ferrumequinum	Rfer002	2011	Ruse	43.7	27.0	Gabarnika	handnet	297/09.03.2011
R. ferrumequinum	Rfer003	2011	Ruse	43.7	27.0	Gabarnika	handnet	297/09.03.2011
R. ferrumequinum	Rfer004	2011	Lovech	43.2	24.4	Parnitzite	mist net	297/09.03.2011
R. ferrumequinum	Rfer005	2011	Lovech	43.2	24.4	Parnitzite	mist net	297/09.03.2011
R. ferrumequinum	Rfer006	2011	Gabrovo	42.4	25.1	Central Balkan	mist net	297/09.03.2011
R. ferrumequinum	Rfer009	2011	Gabrovo	42.4	25.1	Central Balkan	mist net	297/09.03.2011
R. ferrumequinum	Rfer001	2012	Lovech	43.2	24.4	Parnitzite	mist net	465/29.06.2012
R. ferrumequinum	Rfer002	2012	Lovech	43.2	24.4	Parnitzite	mist net	465/29.06.2012
R. ferrumequinum	Rfer004	2012	Lovech	43.2	24.4	Parnitzite	mist net	465/29.06.2012
R. ferrumequinum	Rfer006	2012	Lovech	43.2	24.4	Parnitzite	mist net	465/29.06.2012
R. ferrumequinum	Rfer008	2012	Lovech	43.2	24.4	Parnitzite	mist net	465/29.06.2012
R. ferrumequinum	Rfer001	2014	Ruse	43.5	25.9	Orlova Chuka	harp trap	554/20.01.2014
R. ferrumequinum	Rfer002	2014	Lovech	42.9	24.5	Barima	mist net	554/20.01.2014
R. ferrumequinum	Rfer003	2014	Lovech	42.9	24.5	Barima	mist net	554/20.01.2014
R. ferrumequinum	Rfer004	2014	Lovech	42.9	24.5	Barima	mist net	554/20.01.2014
R. ferrumequinum	Rfer005	2014	Lovech	42.9	24.5	Barima	mist net	554/20.01.2014
R. ferrumequinum	Rfer006	2014	Lovech	42.9	24.5	Barima	mist net	554/20.01.2014
R. ferrumequinum	Rfer007	2014	Lovech	42.9	24.5	Barima	mist net	554/20.01.2014
R. ferrumequinum	Rfer008	2014	Ruse	43.6	25.9	Tabachka	mist net	554/20.01.2014
R. ferrumequinum	Rfer009	2014	Ruse	43.5	25.9	Ostritsa	handnet	554/20.01.2014
R. ferrumequinum	Rfer010	2014	Ruse	43.5	25.9	Ostritsa	handnet	554/20.01.2014
