# Supplementary material for: Bat Species Comparisons Based on External Morphology: A Test of Traditional versus Geometric Morphometric Approaches
Source: PLoS One. 2015 May 12;10(5):e0127043. doi: 10.1371/journal.pone.0127043 (PMC4428882; doi:10.1371/journal.pone.0127043)
Supplement: S2 Table — SS = sum of squares; MS = mean squares; df = degrees of freedom. (PDF) [file pone.0127043.s002.pdf]

S2 Table. Procrustes ANOVA on repeated mesures of shape in the geometric morphometric dataset. SS=sum of squares; MS=mean squares; df=degrees of freedom.

Term	SS	MS	df	F	p	Pillai trace	p
Species	0.06669718	0.00055581	120	5.33	<.0001	3.36	<.0001
Individual	0.22215037	0.00010430	2130	2.97	<.0001	20.65	<.0001
Fixation	0.07783701	0.00003506	2220	9.38	<.0001		
Residual (picture)	0.00325134	0.00000374	870				
