# Supplementary material for: Bat Species Comparisons Based on External Morphology: A Test of Traditional versus Geometric Morphometric Approaches
Source: PLoS One. 2015 May 12;10(5):e0127043. doi: 10.1371/journal.pone.0127043 (PMC4428882; doi:10.1371/journal.pone.0127043)
Supplement: S3 Table — (PDF) [file pone.0127043.s003.pdf]

S3 Table. List of all traditional morphometric variables used for the discriminant analysis and description of how the measures were taken and what general functional importance they have for flight performance.

Measure / ratio	Reference	Refers to method	Measuring method / tool	How measured / computed?	Region on bat	Functional importance for flight performance
handwing length	Norberg & Rayner 1987	methods 1 & 2	Photoshop	length measure	right wing	mainly related to overall size differences: smaller species should generally perform better in confined and cluttered space and larger species should perform poorer [1,2]
armwing length	Norberg & Rayner 1987	methods 1 & 2	Photoshop	length measure	right wing	
handwing area	Norberg & Rayner 1987	methods 1 & 2	Photoshop	area measure	right wing	
armwing area	Norberg & Rayner 1987	methods 1 & 2	Photoshop	area measure	right wing	
wing area	Norberg & Rayner 1987	methods 1 & 2	Photoshop	area measure	wing, body and tail	
wing span	Norberg & Rayner 1987	methods 1 & 2	Photoshop	length measure to body center	right wing and body	
tip length ratio	Norberg & Rayner 1987	methods 1 & 2	computation	ratio: handwing length / armwing length	right wing	ratios related to the wing-tip shape: species with more rounded wing-tips (higher values for wing-tip shape index) should be able to fly more slowly and to be more manoeuvrable which favours flight in cluttered habitat [1,2]
tip area ratio	Norberg & Rayner 1987	methods 1 & 2	computation	ratio: handwing area / armwing area	right wing	
wing tip shape index	Norberg & Rayner 1987	methods 1 & 2	computation	index: tip area ratio / (tip length ratio - tip area ratio)	right wing	
wing loading	Norberg & Rayner 1987	method 1	computation	weight*g / wing area	wing, body and tail	bats with low wing loadings can generally fly at lower speeds than bats with higher wing loadings, for species foraging close to or within vegetation a low wing loading is advantageous [1,2]
relative wing loading	Norberg 1994	method 2	computation	weight*g / wing area*weight^(1/3)	wing, body and tail	
aspect ratio	Norberg & Rayner 1987	methods 1 & 2	computation	wing span^2 / wing area	wing, body and tail	a high aspect ratio indicates narrow wings and a low aspect ratio indicates broader wings, the latter is in combination with low wing loading considered as favourable for flight within cluttered environment [1,2]
tail area	Schmieder et al. 2014	method 2	Photoshop	photoshop area measure	tail	a larger tail membrane can increase manoeuvrability and agility and favours therefore flight in cluttered habitat [2,3]
tail-to-wing area ratio	Schmieder et al. 2014	method 2	computation	tail area / wing area - tail area	wing, body and tail	
forearm length	Dietz et al. 2006	method 3	caliper	length measure folded wing	right wing	mainly related to overall size differences: smaller species should generally perform better in confined and cluttered space and larger species should perform poorer [1,2]
digit 3	Dietz et al. 2006	method 3	TMorphGen6 /Matlab	distance between landmarks 1 and 7	right wing	
digit 5	Dietz et al. 2006	method 3	TMorphGen6 /Matlab	distance between landmarks 7 and 9	right wing	
1st phalanx of 4th finger	Dietz et al. 2006	method 3	TMorphGen6 /Matlab	distance between landmarks 5 and 6	right wing	
2nd phalanx of 4th finger	Dietz et al. 2006	method 3	TMorphGen6 /Matlab	distance between landmarks 4 and 5	right wing	

1. Norberg UM, Rayner JMV. Ecological morphology and flight in bats (Mammalia, Chiroptera) - Wing adaptations, flight performance, foraging strategy and echolocation. Philos Trans R Soc Lond B Biol Sci. 1987; 316: 337-419.

2. Norberg UM.Wing design, flight performance, and habitat use in bats. In: Wainwright PC, Reilly SM, editors. Ecological morphology, integrative organismal biology. Chicago: The University of Chicago Press. 1994. pp. 205-239.

3. Schmieder DA, Zsebők S, Siemers BM. The tail plays a major role in the differing manoeuvrability of two sibling species of mouse-eared bats (Myotis myotis and Myotis blythii). Can J Zool. 2014; 92: 965-977.

4. Dietz C, Dietz I, Siemers BM. Wing measurement variations in the five European horseshoe bat species (Chiroptera : Rhinolophidae). J Mamm. 2006; 87: 1241-1251.
