# Supplementary material for: Bat Species Comparisons Based on External Morphology: A Test of Traditional versus Geometric Morphometric Approaches
Source: PLoS One. 2015 May 12;10(5):e0127043. doi: 10.1371/journal.pone.0127043 (PMC4428882; doi:10.1371/journal.pone.0127043)
Supplement: S4 Table — Rates are expressed as percentages. Species abbreviations as follows: Rhip = R. hipposideros, Rbla = R. blasii, Reur = R. euryale, Rmeh = R. mehelyi, Rfer = R. ferrumequinum. (PDF) [file pone.0127043.s004.pdf]

S4 Table. Cross-validated correct classification rates for each pairwise species comparison across different morphometric methods. Rates are expressed as percentages. Species abbreviations as follows: Rhip = *R. hipposideros* , Rbla = *R. blasii* , Reur = *R. euryale* , Rmeh = *R. mehelyi* , Rfer = *R. ferrumequinum*

Data acquisition	Method	Rhip	Rbla	Reur	Rmeh	Rfer	
Traditional morphometrics	method 1	-					Rhip
		46.2	-				Rbla
Range: 46.2-89.3		89.3	58.6	-			Reur
		88.0	76.9	61.0	-		Rmeh
		88.0	53.8	58.5	65.8	-	Rfer

Data acquisition	Method	Rhip	Rbla	Reur	Rmeh	Rfer	
Traditional morphometrics	method 2	-					Rhip
		92.3	-				Rbla
Range: 58.6-100		100.0	58.6	-			Reur
		100.0	95.8	89.7	-		Rmeh
		100.0	82.1	93.0	73.7	-	Rfer

Data acquisition	Method	Rhip	Rbla	Reur	Rmeh	Rfer	
Traditional morphometrics	method 3	-					Rhip
		76.9	-				Rbla
Range: 69.0-100		100.0	100.0	-			Reur
		100.0	100.0	69.0	-		Rmeh
		70.4	82.1	93.0	95.1	-	Rfer

Data acquisition	Method	Rhip	Rbla	Reur	Rmeh	Rfer	
Geometric morphometrics	method 4	-					Rhip
		84.6	-				Rbla
Range: 84.6 - 100		100.0	100.0	-			Reur
		100.0	85.2	85.7	-		Rmeh
		100.0	100.0	100.0	97.6	-	Rfer
