# Supplementary material for: Bat Species Comparisons Based on External Morphology: A Test of Traditional versus Geometric Morphometric Approaches
Source: PLoS One. 2015 May 12;10(5):e0127043. doi: 10.1371/journal.pone.0127043 (PMC4428882; doi:10.1371/journal.pone.0127043)
Supplement: S5 Table — Species abbreviations as follows: Rhip = R. hipposideros, Rbla = R. blasii, Reur = R. euryale, Rmeh = R. mehelyi, Rfer = R. ferrumequinum. (PDF) [file pone.0127043.s005.pdf]

S5 Table. Correct cross-validated classification rates (CV rate) for the geometric morphometric dataset after dimensionality reduction when performing discriminant analysis on multiple groups (canonical variate analysis = CVA) and in pairwise comparison. Species abbreviations as follows: Rhip = *R. hipposideros* , Rbla = *R. blasii* , Reur = *R. euryale* , Rmeh = *R. mehelyi* , Rfer = *R.ferrumequinum* .

Range of principal components used	All species (CVA)	CV rate for each species in the CVA					Average CV rate - pairwise comparison	Range CV rate - pairwise comparison
	CV rate	Rhip	Rbla	Reur	Rmeh	Rfer		
PC1 - PC2	81.6	83.3	85.7	77.3	70.0	95.2	95.1	76.2 - 100
PC1 - PC3	89.5	100.0	100.0	81.8	85.0	95.2	97.6	85.7 - 100
PC1 - PC10	93.4	100.0	100.0	77.3	100.0	100.0	94.7	69.2 - 100
PC1 - PC17	96.1	100.0	100.0	86.4	100.0	100.0	95.0	61.5 - 100
PC1 - PC25	94.7	100.0	100.0	86.4	95.0	100.0	78.7	37.0 - 100
PC1 - PC30	94.7	100.0	100.0	86.4	95.0	100.0	95.3	84.6 - 100
